# Sleep quality among individuals with ketamine use and the mediating role of craving

**DOI:** 10.1038/s41598-020-77631-9

**Published:** 2020-11-25

**Authors:** Cheng-Fang Yen, Huang-Chi Lin, Chih-Hung Ko, Hung-Chi Wu, Chih-Yao Hsu, Peng-Wei Wang

**Affiliations:** 1 Department of Psychiatry, Faculty of Medicine and Graduate Institute of Medicine, College of Medicine, Kaohsiung Medical University, Kaohsiung, Taiwan; 2 Department of Psychiatry, Kaohsiung Medical University Hospital, 100 Tzyou 1st Road, Kaohsiung, 807 Taiwan; 3 Department of Psychiatry, Kaohsiung Municipal Hsiao-Kang Hospital, Kaohsiung Medical University, Kaohsiung, Taiwan; 4 Department of Addiction Science, Kai-Suan Psychiatric Hospital, Kaohsiung, Taiwan

**Keywords:** Risk factors, Addiction

## Abstract

Sleep problems are common in Taiwan. Poor sleep may be associated with many illnesses, including substance use disorders. Ketamine use disorder has significantly increased in Taiwan in recent years and may lead to physical and cognitive problems. Craving for ketamine is a risk factor for ketamine use and relapse, and poor sleep quality may increase craving. This study aimed to explore the mediating effects of craving on the relationship between poor sleep quality and ketamine use. Demographic data, sleep quality, severity of dependence and craving were recorded for current ketamine users and abstinent ketamine users. Mediation analysis was used to examine the mediating effect of craving on the relationship between poor sleep and ketamine use. This study enrolled 414 current ketamine users with ketamine use disorder, 238 current ketamine users without ketamine use disorder, and 102 abstinent ketamine users with ketamine use disorder. Compared with healthy controls, all ketamine users had poor sleep quality. Poor sleep quality was associated with the initiation of and dependence on ketamine use. Craving can mediate the relationship between poor sleep quality and ketamine use. Poor sleep quality remains a problem for those abstaining from ketamine use. Poor sleep quality in ketamine users is an important issue because it is directly and indirectly through craving associated with ketamine use.

## Introduction

Sleep is an essential physiological process for many vital functions, including restoration of the body, maintenance of energy and modulation of psychological states^1^. Sleep problems are a prevalent public health issue with between 6 and 10% of the population meeting the diagnostic criteria for insomnia^2,3^. Furthermore, approximately one-third of the population experiences sleep problems at some point in their lives^3,4^. Regarding sleep problems, two cross-sectional surveys conducted in 2003 and 2005 showed that the most common sleep problem in Taiwan was insomnia^5,6^. The survey from 2003 revealed that the age-adjusted prevalence of insomnia, defined as difficulty initiating sleep, difficulty maintaining sleep and early morning waking, was 10.3%, which was higher than that in Japan and Korea^5^. Another survey of 36,743 participants aged 18 years and over in 2005 showed that more than 25% had insomnia^6^. A more recent study in Taiwan that used the Pittsburgh Sleep Quality Index (PSQI) to assess sleep quality indicated that up to 46.6% of the participants had poor sleep quality^7^. The authors also argued that the prevalence of poor sleep quality is progressively increasing. All of the above evidence indicates that poor sleep quality is an important health issue in Taiwan.

Ketamine was first introduced and remains widely used as an anesthetic for medical and veterinary use^8^. Reports of recreational use of ketamine can be traced back to 1978^9^, followed by a report of ketamine as a club drug in the 1990s^10^. Consumption of ketamine can result in a variety of health problems, such as cognitive impairment, cystitis, and accidental death^10,11^. Previous studies have documented that ketamine use disorder develops among recreational ketamine users^12,13^. Information regarding the prevalence of this disorder has shown that the population of those using ketamine began to increase worldwide, particularly in Asia, after 2000^14,15^. Compared with drugs such as heroin or amphetamine, ketamine is relatively inexpensive and easy to obtain in Taiwan. Therefore, the culture of drug use in Taiwan underwent a change. A study of drug-related seizures and arrests showed that ketamine has become an increasingly popular illicit substance in Taiwan^16^. A National Household Survey on Health and Substance Abuse conducted in a population aged 12–64 years in Taiwan in 2005 showed that ketamine was the third most commonly abused drug^17^. Knowledge of the underlying factors associated with ketamine use may provide insight into managing individuals suffering from ketamine use disorder because treatment for ketamine use disorder is not yet available^18^.

Craving may result in substance use and relapse of substance abuse in people with substance use disorders^19^. Poor sleep quality may increase craving in people with substance use disorders^20,21^. Poor sleep quality has previously been identified as a risk factor for cocaine, alcohol and opiate use^22–24^. Poor sleep quality is an important health issue in substance users, as complaints regarding sleep are common^25^; however, there has been little study of how poor sleep quality is related to ketamine use disorder and how craving mediates sleep quality and ketamine use. Sleep is a private experience, and the components and importance of sleep quality vary across individuals. Therefore, self-reported evaluations are essential to measure sleep quality. This study aimed to explore the relationships between self-reported sleep quality and ketamine use. We hypothesized that (1) poor sleep quality may be associated with the initiation of and dependence on ketamine use and (2) craving mediates the relationship between sleep quality and ketamine use.

## Method

### Participants

Ketamine users were recruited from the community and from a control environment, namely, from a number of drug rehabilitation centers. The inclusion criteria for participants from the community were as follows: (1) current ketamine use, (2) no other substance use disorders except tobacco use, and (3) no psychiatric diagnoses of schizophrenia, major depressive disorder or bipolar disorder. The inclusion criteria for participants in the control environment were as follows: (1) ketamine use disorder, (2) no other substance use disorders except tobacco use, and (3) no psychiatric diagnoses of schizophrenia, major depressive disorder or bipolar disorder. The healthy controls were those without any substance use disorder or mental illness and were age-, gender- and education-matched to the ketamine users. Initially, all participants, including current/abstinent ketamine users and healthy people, were interviewed by psychiatrists to assess whether or not participants had ketamine use disorder and fulfilled other inclusion criteria. If participants met the inclusion criteria, they underwent interviews and provided baseline data, including age, sex, severity of dependence, craving, sleep quality and mean money spent daily on ketamine use in the preceding week. Informed consent was obtained from all of the participants. The institutional review board of Kaohsiung Medical University approved the study protocol. All experiments were performed in accordance with relevant guidelines and regulations.

### Assessments

#### Visual Analog Craving Scale (VACS)

We used the VACS, modified from previous studies^26,27^, to assess the level of craving in ketamine users from the community and control environment. The VACS consists of the following single question: how much did you crave/desire/want to use ketamine in the preceding week? The level of craving was rated from 0 (not at all) to 100 (very much).

#### Chinese-Mandarin version of the Severity of Dependence Scale (SDS^ch^)

The SDS^ch^, which consists of 5 questions, was used to measure the level of dependence on ketamine in the preceding week^28^. The score on the SDS^ch^ can range from 0 to 15 ^29^.

#### Pittsburgh Sleep Quality Index (PSQI)

We used the PSQI to evaluate sleep quality over the preceding 1-month period. The PSQI is a self-reported questionnaire that is easy to administer^30^. It consists of 19 individual items to generate seven component scores: subjective sleep quality, sleep latency, sleep duration, habitual sleep efficiency, sleep disturbances, use of sleeping medication, and daytime dysfunction. The sum of the seven component scores yields one global score that indicates subjective sleep quality (range 0–21). A higher global score represents poorer subjective sleep quality. The Taiwanese version of the PSQI (PSQI-T) has good validity and reliability^31^. We used a cutoff point of five to indicate poor sleep quality^7^. We calculated rates of poor sleep quality based on PSQI-T > 5.

### Data analysis

Comparison of sleep quality between the healthy controls, current ketamine users without ketamine use disorder, current ketamine users with ketamine use disorder, and abstinent ketamine users with ketamine use disorder was performed using analysis of variance (ANOVA). Tamhane’s T2 was used in the post hoc test to address unequal variances. The Chi-square (χ^2^) test was used to examine differences in categorical variables among groups. Analysis of covariance (ANCOVA) was used to compare sleep quality, craving and level of dependence among groups with the effects of age, gender, education and tobacco use adjusted for. We used a multinomial logistic regression model to explore the associations of poor sleep quality with different types of ketamine use behavior: never used ketamine, nonaddictive use and addictive use. Binary logistic regression was used to examine associations with poor sleep quality in those with addictive use, employing nonaddictive use as a reference. A mediation model was used to explore whether craving was a mediator of the relationship between poor sleep and the amount of money spent on ketamine^32^. The sequential Bonferroni procedure was used to adjust for multiple comparisons^33^.

## Results

There were 844 people enrolled in this study, including 90 healthy controls and 754 ketamine users. Of the ketamine users, 102 (13.52%) had remained abstinent from ketamine for more than three months because they stayed in the control environment, while the others were current ketamine users from the community in South Taiwan. Furthermore, 414 (63.69%) of the current ketamine users from the community fulfilled the criteria for ketamine use disorder as set out in the DSM 5, while the others did not. Among all four groups (healthy controls, abstinent ketamine users with ketamine use disorder, current ketamine users with ketamine use disorder, and current ketamine users without ketamine use disorder), age, gender, education and tobacco use did not differ (Table 1).

**Table 1:** Demographic data, sleep quality and ketamine-related characteristics in healthy controls, current ketamine users and abstinent ketamine users (N = 844).

	Healthy controls (N = 90)	Current ketamine users without ketamine use disorder (N = 238)	Current ketamine users with ketamine use disorder (N = 414)	Abstinent ketamine users with ketamine use disorder in the control environment (N = 102)	F	p value
	N (%) Mean (SD)	N (%) Mean (SD)	N (%) Mean (SD)	N (%) Mean (SD)		
Gender (male)^a^	80 (88.89)	203 (86.02)	353 (85.27)	91 (89.22)		0.651^d^
Age (years)^b^	25.67 (5.14)	24.23 (5.05)	25.35 (5.95)	25.58 (6.79)	2.59	0.051^f^
Tobacco use disorder^a^	48 (53.33)	123 (52.12)	242 (58.45)	53 (51.96)		0.357^e^
Education (years)^b^	11.62 (2.10)	11.42 (2.57)	11.35 (2.21)	11.22 (2.33)	0.53	0.664
Sleep quality^a,c^	4.61 (3.17)	6.13 (2.63)	8.03 (3.47)	6.84 (3.90)	33.99	< 0.001^g^
Rate of poor sleep^a^	43 (47.77)	168 (71.19)	334 (80.68)	74 (72.55)		< 0.001
Craving^b,h^		3.41 (8.95)	15.79 (22.76)	15.98 (25.52)	31.82	< 0.001^g^
Severity of ketamine use^b,i^		0.64 (0.84)	4.87 (2.23)	5.37 (4.77)	251.60	< 0.001^g^
Money spent on ketamine^b^		75.10 (303.06)	494.78 (1146.78)			< 0.001^j^

^a^Values are presented as N (%); ^b^values are presented as mean (SD); ^c^sleep quality was measured using the PSQI; ^d^analyzed by chi-square tests with female gender as the reference; ^e^analyzed by chi-square tests with participants without tobacco use disorder as the reference; ^f^analyzed by ANOVA; ^g^analyzed by ANOVA while controlling for the effects of age, gender, education and tobacco use disorder; ^h^measured by the VACS; ^i^measured by the SDS; ^j^analyzed by *t* test.

There were significant differences in craving for ketamine and the severity of dependence among the current ketamine users without ketamine use disorder, current ketamine users with ketamine use disorder, and abstinent ketamine users with ketamine use disorder after adjusting for the effects of age, gender, education and tobacco use. In particular, the current and abstinent ketamine users with ketamine use disorder had higher levels of craving and a greater severity of dependence than the ketamine users without ketamine use disorder (craving: p_current ketamine users with ketamine use disorder_ < 0.001 and p_abstinent ketamine users_ < 0.001; severity of dependence: p_current ketamine users with ketamine use disorder_ < 0.001 and p_abstinent ketamine users_ < 0.001). The current ketamine users with ketamine use disorder did not differ in terms of the level of craving or the severity of dependence from the abstinent ketamine users with ketamine use disorder. The mean amount (SD) of money spent on ketamine was greater in the current ketamine users with ketamine use disorder than in those without ketamine use disorder (t = 506.54, p < 0.001).

Mean PSQI scores differed significantly among the healthy controls, current ketamine users with/without ketamine use disorder, and abstinent ketamine users after controlling for the effect of age and gender. Post hoc analysis showed that the healthy controls had the lowest mean PSQI score (p_current ketamine users without ketamine use disorder_ = 0.001; p_current ketamine users with ketamine use disorder_ < 0.001 and p_abstinent ketamine users_ < 0.001), while the current ketamine users with ketamine use disorder had the highest (p_current ketamine users without ketamine use disorder_ < 0.001 and p_abstinent ketamine users_ = 0.007). The current ketamine users without ketamine use disorder and the abstinent ketamine users did not significantly differ in terms of mean PSQI scores (p = 0.551). The rates of poor sleep in healthy controls, current ketamine users with/without ketamine use disorder and abstinent ketamine users significantly differed. The rate of poor sleep was significantly higher in the current ketamine users with ketamine use disorder than in the current ketamine users without ketamine use disorder (χ^2^ = 11.84, p = 0.001) and the abstinent ketamine users (χ^2^ = 7.30, p = 0.007). The rate of poor sleep in the current ketamine users without ketamine use disorder did not differ from that in the abstinent ketamine users (χ^2^ = 0.001, p = 0.973).

Multinomial logistic regression analysis showed that sleep quality was significantly associated with the status of ketamine use (Table 2). Poor sleep quality was significantly associated with current ketamine use with and without ketamine use disorder compared with the healthy controls. Furthermore, binary logistic regression in the current ketamine users indicated that poor sleep quality was a risk factor for ketamine use disorder. In addition, both sleep quality and craving for ketamine were associated with the amount of money spent on ketamine in current ketamine users with ketamine use disorder. Mediation analysis showed that poor sleep had an indirect effect via craving on the amount of money spent on ketamine (Table 3).

**Table 2:** Associations between sleep quality and ketamine use status in community ketamine users according to multinomial and binary logistic regression.

	Model I^a^				Model II^b^	
	Odds ratio for current ketamine users without ketamine use disorder (95% CI)	p	Odds ratio for current ketamine users with ketamine use disorder (95% CI)	p	Odds ratio for current ketamine users with ketamine use disorder (95% CI)	p
Gender^c^	0.86 (0.40, 1.87)	0.863	0.82 (0.38, 1.75)	0.606	1.03 (0.63, 1.67)	0.910
Age (years)	0.98 (0.94, 1.03)	0.461	0.98 (0.94, 1.03)	0.461	0.99 (0.96, 1.02)	0.609
Education (years)	0.95 (0.86, 1.06)	0.388	0.94 (0.84, 1.04)	0.229	0.99 (0.92, 1.06)	0.695
Tobacco use disorder^d^	0.97 (0.58, 1.60)	0.895	0.71 (0.43, 1.18)	0.187	1.39 (0.98, 1.97)	0.062
Sleep quality^d^	1.23 (1.12, 1.36)	< 0.001	1.49 (1.35, 1.64)	< 0.001	1.22 (1.16, 1.30)	< 0.001

^a^Multinomial logistic regression using healthy controls as the reference group; ^b^binary logistic regression using current ketamine users without ketamine use disorder as the reference group; ^c^female as the reference; d: measured by the PSQI; ^d^participants without tobacco use disorder as the reference.

**Table 3:** Associations of craving and sleep quality with money spent on ketamine in current ketamine users with ketamine use disorder.

Outcome variable for the model	Model without mediation analysis		Model with mediation analysis			
	Money spent on ketamine		Craving		Money spent on ketamine	
	Coefficient	p	Coefficient	p	Coefficient	p
Gender^a^	− 24.63	0.862	− 1.20	0.667	57.03	0.634
Age (years)	− 13.47	0.122	− 0.28	0.101	− 5.84	0.429
Education (years)	6.92	0.763	0.58	0.202	− 8.72	0.655
Tobacco use disorder^b^	− 9.80	0.925	− 2.03	0.321	64.70	0.462
Severity of ketamine use^c^	54.88	0.020	2.00	< 0.001	0.51	0.980
Craving^d^					27.09	< 0.001
Sleep quality^e^	162.92	< 0.001	2.85	< 0.001	85.58	< 0.001

^a^Female as the reference; ^b^no tobacco use disorder as the reference; ^c^measured by the SDS; ^d^measured by the VACS; ^e^measured by the PSQI.

## Discussion

This study found that (1) current ketamine users with and without ketamine use disorder and abstinent ketamine users had higher mean PSQI scores and higher rates of poor sleep quality than healthy controls; (2) poor sleep quality was associated with current nonaddictive or addictive ketamine use; and (3) craving may have mediated the relationship between sleep quality and money spent on ketamine. Poor sleep quality is a mental health issue not only for current ketamine users without ketamine use disorder but also for current ketamine users with ketamine use disorder compared to healthy people. Furthermore, the participants who had abstained from ketamine for more than three months also had sleep problems, which indicated that sleep problems were more prevalent in ketamine users regardless of the status of ketamine use than in healthy people. Therefore, mental health professionals should evaluate sleep quality when helping current and abstinent ketamine users.

Strong evidence has demonstrated the role of poor sleep quality in substance users in relation to an increased risk of relapse^34,35^. Therefore, poor sleep quality can be an indicator of a higher probability of relapse in abstinent ketamine users. In addition, the abstinent ketamine users with ketamine use disorder had a lower mean PSQI score and a lower rate of poor sleep quality than the current ketamine users with ketamine use disorder. This result was in line with previous studies involving cocaine and alcohol users, which showed that abstinence from these substances cannot completely reverse sleep problems^23,36^. However, studies on sleep quality in abstinent ketamine users are scarce. Due to the nature of the observational study, these results provided only preliminary evidence that ketamine users with ketamine use disorder do not experience complete remission in sleep quality after stopping ketamine use. Many factors may contribute to the presence of sleep problems in abstinent ketamine users. For example, sleep problems may have preceded the development of substance use^25^, and chronic substance use makes sleep problems worse^37^. Therefore, abstinence from substance use only partially reverses sleep problems. It would be worthwhile to explore the exact factors that contribute to poor sleep in abstinent ketamine users.

The present study found that poor sleep quality was associated with current ketamine use. Poor sleep was also associated with ketamine use disorder in current ketamine users. Information regarding how poor sleep is related to the status of ketamine use is limited. Roehr et al. reviewed previous studies on substances other than ketamine and argued that poor sleep may result in the initiation and maintenance of drug use^25^. Evidence has also indicated that sleep problems increased the risk of developing substance use disorders^24,38^. Our results provide evidence that poor sleep is also an indicator associated with the initiation of and dependence on ketamine use.

The analysis without mediation indicated that poor sleep was related to the amount of money spent on ketamine, which is an indicator of the amount of ketamine being used. The mediation analysis showed that the relationship between poor sleep and the amount of money spent on ketamine involves at least two pathways. One is a direct pathway, while the other is that poor sleep is related to money spent on ketamine by craving. Previous studies with substances other than ketamine demonstrated that poor sleep was positively associated with craving in substance users^39,40^. Our findings also demonstrated that poor sleep was positively associated with craving for ketamine, indicating a potential role of craving in mediating the relationship between sleep problems and ketamine use.

In conclusion, our findings raised some important implications for those with ketamine use disorder. First, sleep problems can persist in abstinent ketamine users. Second, sleep quality is important to ketamine use because poor sleep quality may be associated with increased risks of initiation and dependence. Third, the association between sleep problems and the amount of ketamine used may be indirect with craving as a mediator. Despite the current study having examined the associations between sleep quality, craving, and ketamine-related outcomes, there were limitations inherent in this investigation. First, we relied on self-reported data to measure sleep quality and ketamine-related outcomes. It is possible that these measurements contained errors and therefore may have biased our results. Objective sleep quality assessments by sleep monitoring devices can be used to reduce bias. Further study on the association between objective sleep qualities and ketamine use is warranted. Second, the use of cross-sectional data limited our ability to draw causal inferences regarding the determinants of poor sleep quality and ketamine use/outcomes. Third, the participants were voluntarily recruited. We enrolled participants meeting rigorous criteria and included variables in the analysis to reduce selection bias^41,42^. Fourth, fewer female participants may also limit the generalizability to the female population. Fifth, craving is a dynamic phenomenon. This may have contributed to levels of craving that were not high, because we asked about the average level of craving for the past week not the peak level of craving. Using the VACS to assess the mean level during the past week is feasible in studies of substance use disorder^43^. Sixth, fewer healthy controls were enrolled because we matched the controls to ketamine users by gender, age and education. Fewer healthy controls may have reduced the power of the analysis^44^. Enrolling more healthy controls while matching the low education level of the ketamine users is not easy in Taiwan because most healthy controls have higher education levels. Therefore, future studies with matched groups in a 1:1 ratio is warranted. This study was among few that have explored the relationship between sleep quality, craving and ketamine-related outcomes. By obtaining a better understanding of the role of sleep quality in ketamine users in relation to ketamine-related outcomes, we can make advances in devising more effective, personalized ketamine cessation interventions and relapse prevention strategies. Future research on the efficacy of clinical interventions designed to improve sleep quality in ketamine users is warranted to address cravings and promote cessation.
